# Society Meetings

**Published:** 1893-04

**Authors:** 


### Society Meetings.

THE nineteenth annual meeting of the American Neurological Association will be held in Saratoga, N. Y., July 25, 26, and 27, 1893.

THE next meeting of the Buffalo Microscopical Club occurs Monday, April 10, 1893, 8.15 P. M., at the Buffalo Library building. Professor W. W. Rowlee, of Cornell University, will read a paper on A Study in the Minute Structure of the Organs of Aquatic Plants.

THE forty-fourth annual session of the Medical Association of Georgia will be held in Americus on April 19, 20 and 21st. President, A. A. Smith, M. D., Hawkinsville; Vice-Presidents, Geo. J. Grimes, M. D., Columbus, and Robt. H. Taylor, M. D., Griffin; Secretary, Dan. H. Howell, M. D., Atlanta; Treasurer, E. C. Goodrich, M. D., Augusta.

THE American Association of Obstetricians and Gynecologists will hold its sixth annual meeting in Detroit, Mich., Thursday, Friday, and Saturday, June 1, 2, and 3, 1893, under the Presidency of Dr. Lewis S. McMurtry, of Louisville, Ky.

It is expected that this will be one of the most interesting meetings in the history of the Association, and all members of the profession interested are cordially invited to attend.

*By Order of the Executive Council.*

THE French Society of Electropathy is about to manage a yearly exhibition, which will take place the Friday and Saturday of Easter week in 1893.

This exhibition will be held in the "Laboratoire de physique de la Faculté de Médecine" in Paris, and will include the instruments employed in electropathy, as well as demonstrations concerning electric methods, drawings, etc.

The organizing committee is represented by Prof. Gariel, Drs. Tripier, Gautier, Vogt, and M. Gaiffe, constructor.

Doctors and constructors are invited to call from this day upon Dr. Vogt, 28 rue Saint-Lazare, Paris, for information.

---

American Surgical Association.—The preliminary programme for the meeting to be held at Buffalo, N. Y., May 30, 31, and June 1, 1893, is a list of the subjects which have been selected for special consideration at the next meeting, with the names of the Fellows who have promised to prepare the opening papers upon the several subjects, and of those who are expected to open and take part in the discussions:

President's Address.

The Modern Treatment of Compound Fractures, by Dr. Nicholas Senn, Chicago; discussion by Drs. Roswell Park and F. S. Dennis.

Hypertrophies and Degenerations of Cicatrices and Cicatricial Tissue, by Dr. J. Collins Warren, Boston; discussion by Drs. C. H. Mastin, G. R. Fowler, and W. H. Carmalt.

Surgery of the Gall Bladder, by Dr. M. H. Richardson, Boston; discussion by Drs. J. Ewing Mears, A. Vander Veer, W. H. Carmalt, and Theo. A. McGraw.

Surgery of the Rectum, by Dr. A. G. Gerster, New York; discussion by Drs. L. S. Pilcher, H. H. Mudd, and L. McLane Tiffany.

Surgical Treatment of Cervical, Thoracic, and Abdomina Aneurism, by Dr. C. B. Nancrede, Ann Arbor; discussion by ——— ———

Surgery of the Prostate, by Dr. J. William White, Philadelphia; discussion by Drs. Hunter McGuire, T. F. Prewitt, R. F. Weir, and F. H. Gerrish.

Treatment of Carbuncle, by Dr. F. Lange, New York; discussion by Drs. Robert Abbe, J. B. Roberts, and J. S. Wight.

In addition to the above specially-selected subjects, the following papers have been offered:

Unreduced Dislocations of the Astragalus, by Dr. Stephen Smith, New York.

Fellows who propose to present volunteer papers are requested to send to either of the undersigned the titles of their proposed papers as soon as possible, in order that they may be properly classified and arranged for the final programme.

Fellows are also requested to notify the Chairman of the Committee whether they will take part in the discussion upon any of the special subjects given in the above list.

NICHOLAS SENN, *President*,
J. R. WEIST, *Secretary*,
J. EWING MEARS, *Recorder*,
F. S. DENNIS,
JOHN S. BILLINGS,
*Business Committee.*
R. PARK,
*Chairman Com. of Arrangements.*

---

PAN-AMERICAN MEDICAL CONGRESS.—*Section on Hygiene, Climatology and Demography.*—Persons proposing to present papers before this Section are requested to communicate with either of the undersigned *immediately*, that titles of subjects may be properly classified for the programme of the proceedings of the Congress. The only limitation as to subject matter is that it shall have a sanitary, climatological, or statistical bearing. Members of the Section on State Medicine of the American Medical Association, the American Public Health Association, the American Climatological Association, the American Academy of Medicine, and of State and Municipal Boards of Health are especially invited to contribute the results of their several experiences. The languages of the Congress being Spanish, Portuguese, French, and English, papers may be presented in either, to be translated in the others, for which reason their text should be in the hands of the secretaries at the earliest possible date.

ALBERT L. GIHON, M. D., *President*,
145 East 21st street, New York City.
PEDRO JOSE SALICRUP, M. D., *Secretary* (Spanish),
129 East 17th street, New York City.
PETER H. BRYCE, M. D., *Secretary* (English),
Toronto, Canada.

---

THE Section on Laryngology and Rhinology of the Pan-American Medical Congress is now thoroughly organized, with secretaries in all the countries of South America as well as in the United States and Canada.

The President, Dr. E. Fletcher Ingals, of Chicago, is making a thorough canvass to secure a large number of good papers for the Section, and aided as he will be by the able Secretaries, Drs. Murray and y Alonso, and the corps of Honorary Presidents, he feels assured of the success of this department of the Congress. The Honorary Presidents are:

Drs. Harrison Allen, Philadelphia; Franke H. Bosworth, New York; J. Solis Cohen, Philadelphia; D. Bryson Delavan, New York; J. F. Dixon, Portland, Ore.; Stephen Dodge, Halifax, N.S.; W. C. Glasgow, St. Louis; Frederick I. Knight, Boston; Geo. M. Lefferts, New York; Alvaro Ledan, Villa Clara, Cuba; John N. Mackenzie, Baltimore; David Matto, Lima, Peru; P. Emelio Petit, Santiago, Chili; John O. Roe, Rochester, N. Y.; Federico Semeleder, City of Mexico, Mex.; Chas. E. Sajous, Paris, France.

The Secretaries for foreign countries are: Drs. Ovejero, (Piedad 22,) Buenos Ayres, Argentine Republic; H. Guedes de Mello, Rio de Janeiro, U. S. of Brazil; G. W. Major, Montreal, Canada; Felix Campuzano, (Virtudes 33,) Havana, Cuba; Luis Fonnegra, (Calle 10, Numero 263,) Bogota, Republic of Columbia; Fabricio Uribe, Guatemala City, Guatemala; Henri Goulden McGrew, Honolulu, Hawaii; Angel Gavino, (Cocheros 15,) City of Mexico, Mex.; J. Midence, Leon, Nicaragua; Eugenios Cassanello, (San José 119,) Montevideo, Uruguay; Napoleon F. Cordero, Merida, Venezuela.

All physicians interested in this Section are requested to correspond with the secretaries for the United States.

| | |
|---|---|
| Dr. J. Maron y Alonso, | Dr. T. Morris Murray, |
| (Spanish speaking), | (English speaking), |
| Las Vegas, N. M. | Washington, D. C. |

---

The Section of Gynecology and Abdominal Surgery of the Pan-American Medical Congress of 1893, has been completely organized, with the following list of officers:

Executive President, Dr. William Warren Potter, 284 Franklin street, Buffalo, N. Y.; English-speaking Secretary, Dr. Brooks H. Wells, 71 W. Forty-fifth street, New York; Spanish-speaking Secretary, Dr. E. W. Cushing, 168 Newbury street, Boston, Mass., together with the following list of honorary presidents:

Dr. Rafael Benavides, Lima, Peru; Dr. Young H. Bond, St. Louis; Dr. Domingo F. Cubas, Havana, Cuba; Dr. Clinton Cushing, San Francisco; Dr. Wm. E. B. Davis, Birmingham, Ala.; Dr. Christian Fenger, Chicago, Ill.; Dr. Frank P. Foster, New York; Dr. Thos. H. Hawkins, Denver; Dr. Wm. D. Haggard, Nashville; Dr. Edward W. Jenks, Detroit; Dr. Joseph Taber Johnson, Washington; Dr. Ernst S. Lewis, New Orleans; Dr. Andres Lopez Martinez, Tegucigalpa, Honduras; Dr. Richard B. Maury, Memphis; Dr. Thomas E. McArdle, Washington; Dr. Lewis S. McMurtry, Louisville; Dr. Roberto Moericke, Santiago, Chile; Dr. Robt. T. Morris, New York City; Dr. Paul F. Mundé, New York; Dr. Joseph Price, Philadelphia; Dr. Charles A. L. Reed, Cincinnati; Dr. John C. Reeve, Dayton, O.; Dr. José Manuel de los Rios, Caracas, Venezuela; Dr. George H. Rohé, Catonsville, Md.; Dr. James F. W. Ross, Toronto, Canada; Dr. Albert Vander Veer, Albany; Dr. Milo B. Ward, Topeka; Dr. Henry P. C. Wilson, Baltimore; Dr. Nicolás San Juan, City of Mexico, Mexico.

The following is a list of the Advisory Council:

Dr. A. H. Cordier, Kansas City, Mo.; Dr. J. H. Carstens, Detroit, Mich.; Dr. Edwin Walker, Evansville, Ind.; Dr. Rufus B. Hall, Cincinnati; Dr. X. O. Werder, Pittsburg, Pa.; Dr. A. F. Currier, New York, N. Y.; Dr. Robert T. Morris, New York, N. Y.; Dr. F. H. Davenport, Boston, Mass.; Dr. Ely Van de Warker, Syracuse, N. Y.; Dr. George R. Dean, Spartanburg, S. C.; Dr. George C. Jarvis, Hartford, Conn.; Dr. Henry T. Byford, Chicago; Dr. George F. French, Minneapolis, Minn.; Dr. J. F. Y. Paine, Galveston, Texas.

It is desirable that all those who propose to read papers before this Section should nominate their titles to the secretaries above named at an early day, as the regulations require that abstracts of such papers shall be in the hands of the Secretary-General not later than July 10, 1893.

All members of the profession of medicine interested are earnestly and cordially invited to attend the meetings of the section to be held in Washington, Tuesday, Wednesday, Thursday, and Friday, September 5, 6, 7, and 8, 1893.

*By Order of the Executive President.*

BROOKS H. WELLS, *English-speaking Secretary.*

E. W. CUSHING, *Spanish-speaking Secretary.*

